# Dissemination mechanisms of NDM genes in hospitalized patients

**DOI:** 10.1093/jacamr/dlab032

**Published:** 2021-03-30

**Authors:** Yuting Zhai, Shinyoung Lee, Lin Teng, Zhengxin Ma, Nicole B Hilliard, Robert J May, Scott A Brown, Fahong Yu, Kathryn E Desear, Kartik Cherabuddi, Kenneth H Rand, J Glenn Morris, Nicole M Iovine, KwangCheol C Jeong

**Affiliations:** 1 Emerging Pathogens Institute, University of Florida, Gainesville, FL, USA; 2 Department of Animal Sciences, Institute of Food and Agricultural Sciences, University of Florida, Gainesville, FL, USA; 3 Infection Control, University of Florida Health/Shands Hospital, Gainesville, FL, USA; 4 ICBR, University of Florida, Gainesville, FL, USA; 5 Department of Pharmacy, University of Florida Health/Shands Hospital, Gainesville, FL, USA; 6 Division of Infectious Diseases and Global Medicine, College of Medicine, University of Florida, Gainesville, FL, USA

## Abstract

**Background:**

NDM-producing Enterobacteriaceae are a major clinical concern worldwide. We characterized NDM-positive pathogens isolated from patients and assessed the dissemination patterns of the *bla*_NDM_ genes in a hospital setting.

**Methods:**

Eleven NDM-positive Enterobacteriaceae (three *Enterobacter hormaechei*, six *Klebsiella pneumoniae* and two *Escherichia coli*) were isolated from nine patients over a 1 year period. Antimicrobial susceptibility was assessed by MICs. A combination of short- and long-read WGS was used for genome analysis. Clinical treatment history of patients was linked with genetic features of individual isolates to investigate the dissemination patterns of the *bla*_NDM_ genes and NDM-positive strains.

**Results:**

*bla*_NDM_ in clonal *K. pneumoniae* were transmitted between two patients. In other instances, an identical IncC plasmid encoding NDM-1 was transmitted between *E. coli* and *K. pneumoniae* isolated from the same patient, and an IncX3 plasmid, carrying *bla*_NDM-1_ or *bla*_NDM-5_, was harboured in non-clonal *E. hormaechei*. Varying patterns of IS elements were identified as a critical transmission mechanism in association with *bla*_NDM_ genes.

**Conclusions:**

Multiple transmission patterns were identified in hospitalized patients, including dissemination of clonal bacterial strains carrying resistance genes and horizontal transfer of resistance genes among divergent bacterial strains. Controlling spread of NDM is complex: while attention to standard infection control practices is critically important, this needs to be matched by aggressive efforts to limit unnecessary antimicrobial use, to minimize the selection for and risk of transfer of ‘high mobility’ resistance genes among Enterobacteriaceae.

## Introduction

Carbapenem-resistant Enterobacteriaceae (CRE) were identified as an ‘urgent threat’ in the 2019 Antibiotic Resistance Threats Report by CDC.^1^ In 2017, CDC estimated that there were 13 100 cases, 1100 deaths and $130 million in healthcare costs caused by CRE.^1^ CRE have two subsets: carbapenemase-producing Enterobacteriaceae (CPE) and non-carbapenemase-producing Enterobacteriaceae. As CPE spread quickly in hospital settings, they are a rising clinical concern globally.^1–4^ Carbapenemases produced by Enterobacteriaceae are divided into three classes of β-lactamase (class A, B and D), based on their molecular structure.^5^^,^^6^ The five common carbapenemases include *Klebsiella pneumoniae* carbapenemase (KPC) belonging to class A, New Delhi metallo-β-lactamase (NDM), Verona integron-encoded metallo-β-lactamase (VIM) and imipenemase (IMP) belonging to class B, and oxacillinase-48 (OXA-48) belonging to class D.^6^

Due to its unprecedented speed of spread, NDM has drawn global attention in the last decades.^7^ NDM-1 was first identified in a *K. pneumoniae* strain isolated from a patient from New Delhi, India, who was hospitalized in Sweden in 2008.^8^ By 2019, a total of 24 NDM variants (NDM-1 to NDM-24) were identified in various bacterial species globally.^9^ Among the NDM-positive Enterobacteriaceae, *K. pneumoniae* account for more than half of the total number, followed by *Escherichia coli* and *Enterobacter cloacae* complex (which includes *Enterobacter hormaechei* isolated in this study).^9^ Given the ability to hydrolyse most β-lactams (with the exception of monobactams), infections with NDM-producing strains have very limited therapeutic options, with a high associated mortality rate among infected patients.^10^^,^^11^ However, limited data are available on the treatment of infections caused by NDM-bearing microorganisms using existing antibiotic options. In addition, much of the newer antimicrobials and experimental pipeline is unable to inhibit this group of carbapenemases.

The NDM-encoding genes, *bla*_NDM-1_ to *bla*_NDM-24_, have been reported in conjugative plasmids belonging to several incompatibility groups (Inc).^9^ In addition to plasmids, other mobile genetic elements (MGEs), such as transposons, IS elements, and integrons, have also been associated with NDM mobilization.^12^^,^^13^ However, lower resolution techniques such as PCR-based targeted partial genotyping or short-read WGS based unclosed genome often fail to provide a comprehensive picture of mechanisms underlying NDM transmission.^14^^,^^15^ Here, we focus on routes of NDM transmission over time in a major, tertiary-care referral hospital. By using a combination of short- and long-read WGS we provide insights into transmission mechanisms, highlighting areas of concern in trying to control nosocomial spread of this potentially dangerous gene complex.

## Methods

### MICs and bacterial isolation

Presence of CRE and associated MICs were identified using the Vitek^®^2 Microbial Identification System (bioMérieux) in the University of Florida (UF) Health Shands Hospital Clinical Microbiology Laboratory. MICs were subsequently confirmed via Etest (bioMérieux). Organisms were further analysed via the Xpert^®^ Carba-R (Cepheid) to detect the presence of carbapenemase genes.^16^ As part of standard daily hospital infection control activities, all Gram-negative bacterial isolates found by the hospital microbiology laboratory to have resistance to carbapenem were identified through an automated infection control/laboratory surveillance system, and basic data on patient location and movement during hospitalization were collected. This resulted in identification of 11 NDM-carrying strains across a 1 year time period; sequence data were obtained for all 11 strains, as described below. A subset of patients infected with NDM strains were enrolled in an institutional review board-approved study at the University of Florida that permitted collection of additional clinical and epidemiological data from patients infected with antimicrobial-resistant bacterial strains. Informed consent was not required for the use of de-identified samples.

### WGS and assembly

For Illumina WGS and assembly, DNA of the 11 strains identified as carrying NDM was extracted using the DNeasy blood and tissue kit (Qiagen, Valencia, CA, USA) following the protocol for Gram-negative bacteria. DNA libraries were constructed using the Nextera XT sample preparation kit (Illumina, San Diego, CA, USA) following the protocol from manufacturer. The sequencing steps and assembly settings were performed as described previously.^17^

For PacBio sequencing, DNA was extracted from six selected isolates using the Puregene Yeast/Bact. Kit B (Qiagen) and was cleaned up using the DNeasy PowerClean Cleanup Kit (Qiagen), following the protocols for Gram-negative bacteria. Then, the DNA samples were sent to the Interdisciplinary Center for Biotechnology Research (ICBR) of University of Florida for PacBio Sequel sequencing. The raw reads generated from the PacBio Sequel I sequencing system were demultiplexed with the PacBio SMRT Analysis (7.0.1.66974). The sub-reads for each sample were assembled by the HGAP4 (hierarchical genome assembly process) and Canu v2.0 with optimized parameters to generate *de novo* genome chromosomes and plasmids.^18^ Both assemblers filtered the sequencing data to remove SMRTbell adapter sequences and recover high-quality genomic content. The initial assemblies were further checked and validated with the samtools, FASTX-toolkit and R-based scripts developed in house at ICBR. The validated assemblies were imported into SMRT Link for subsequent polishing with the Resequencing Analysis to attain a higher base quality. The finalized genomes and plasmids were circularized using the Circlator tool.^19^ Assembled genomes were deposited in NCBI (Table S1, available as Supplementary data at *JAC-AMR* Online).

### Phylogenetic tree analysis and antibiotic resistance gene (ARG) identification

Phylogenetic trees were generated using Illumina sequencing results for each species separately, using Parsnp (https://harvest.readthedocs.io/en/latest/) with default settings, based on the core-genome SNPs. The reference strains for each species were chosen by Parsnp randomly, and KCJ3K13, KCJ3K293 and KCJ3K291 were selected as the reference strains for *E. hormaechei*, *K. pneumoniae* and *E. coli*, respectively. SNPs in each phylogenetic clade were calculated using NCBI Pathogen Detection (https://www.ncbi.nlm.nih.gov/pathogens/).

To identify the ARGs, the genomic sequences were compared with the reference sequences in the Comprehensive Antibiotic Resistance Database (CARD 3.0.8) using the Resistance Gene Identifier (RGI 5.1.0).^20^ Bacterial DNA sequences were submitted to the web portal of CARD with the selected parameters of ‘Perfect and Strict hits only’, ‘Include nudge’ and ‘Low quality/coverage’.

### Plasmid typing and NDM genetic environment

Plasmids were assigned to incompatibility groups using PlasmidFinder 2.1 (https://cge.cbs.dtu.dk/services/PlasmidFinder/). The completed circular genome maps of chromosomal DNA and plasmids were generated using BLAST Ring Image Generator (BRIG).^21^ The annotations for the genome mapping and generic environments of *bla*_NDM_ flanking genes were acquired from Prokka and the NCBI Prokaryotic Genome Annotation Pipeline (PGAP).^22^

### Bacterial conjugation

The conjugation experiment was performed between *K. pneumoniae* KCJ3K292 and *E. coli* XL1-Blue (tetracycline-resistant strain). Overnight cultures of *K. pneumoniae* KCJ3K292, harbouring NDM-encoding-plasmid pKC148K (donor) and *E. coli* XL1-Blue (recipient) were combined (3:1 ratio). The combined bacteria were conjugated for 20 h on tryptic soy agar (TSA) at 25 °C and *E. coli* transconjugants were selected on TSA plates containing tetracycline (10 mg/L) and meropenem (16 mg/L). Total recipients used for conjugation were counted by selecting on TSA containing only tetracycline (10 mg/L). Conjugation frequency was calculated by dividing the number of transconjugants by the number of recipients used.

## Results

The 11 NDM-producing Enterobacteriaceae strains (three *E. hormaechei*, six *K. pneumoniae* and two *E. coli*), were isolated over a 1 year period from nine patients from UF Health Shands hospital. All nine patients were discharged alive after the admission during which the NDM-bearing organism was identified. The median age was 52 years (range 2–67), five were male (56%) and four were female (44%). Four patients (44%) were admitted from a congregate setting or another hospital. Two patients were admitted in septic shock, and four were admitted with necrotizing fasciitis. Patients were relocated between North Tower (NT), South Tower (ST), and East Tower (ET) of the hospital for treatment. Length of hospitalization ranged from 11 days to more than 9 months. The average number of days that elapsed between admission and isolation of the NDM-bearing organism was 14 (range 0–38). Five of the nine (56%) had been hospitalized in the year prior to isolation of the NDM-bearing organism, and of these the number of prior admissions ranged from one to five. Of the 11 isolates, 6 were obtained from wound cultures from four patients, 3 isolates were from urine, 1 was from ascitic fluid and 1 was from blood. All patients were initially treated with cefepime, and therapy was changed to agents active against the NDM-bearing organism(s) once they were identified in eight of the nine patients (one result was obtained after the patient was discharged to the sending facility) (Table S2).

To investigate the antibiotic resistance profile, the MIC was obtained using standard Vitek^®^2 susceptibility cards (Table 1). Twenty-three antibiotics and combinations, belonging to 12 antibiotic classes, including aminoglycoside, penicillin, penicillin/β-lactamase inhibitor, cephalosporin, fluoroquinolone, carbapenem, cephalosporin/β-lactamase inhibitor, sulphonamide, glycylcycline, polymyxin, tetracycline and nitrofurantoin (urine isolates only) were tested (Table 1). All strains were MDR in which *K. pneumoniae* KCJ3K307 was resistant to 14 antibiotics, while *K. pneumoniae* KCJ3K65 was resistant to the least number of antibiotics (*n *=* *12). All isolates were resistant to piperacillin/tazobactam, cephalosporin class drugs and meropenem. All isolates were susceptible to amikacin.

**Table 1.** MIC (mg/L) of NDM strains isolated from hospitalized patients

Strains	*E. hormaechei*			*K. pneumoniae*						*E. coli*	
	KCJ3K13	KCJ3K19	KCJ3K22	KCJ3K53	KCJ3K65	KCJ3K270	KCJ3K292	KCJ3K293	KCJ3K307	KCJ3K291	KCJ3K426
Patients	A	B	G	E	F	H	C	C	D	C	I
Specimen source	wound	wound	urine	blood	urine	urine	wound	wound	wound	wound	ascitic fluid
Isolated date	3 Apr 2019	6 Mar 2019	2 Nov 2018	4 Jul 2018	8 Aug 2018	15 Apr 2019	6 May 2019	6 May 2019	4 Jun 2019	6 May 2019	13 Jul 2019
Antimicrobial											
amikacin	≤2 (S)	4 (S)	≤2 (S)	≤2 (S)	≤2 (S)	≤2 (S)	≤2 (S)	≤2 (S)	≤2 (S)	4 (S)	≤2 (S)
gentamicin	≤1 (S)	8 (I)	≥16 (R)	≥16 (R)	≥16 (R)	≥16 (R)	≥16 (R)	≥16 (R)	≥16 (R)	≥16 (R)	≥16 (R)
tobramycin	≤1 (S)	≥16 (R)	8 (I)	8 (I)	8 (I)	≥16 (R)	8 (I)	8 (I)	≥16 (R)	≥16 (R)	8 (I)
ampicillin	NA	NA	NA	≥32 (R)	≥32 (R)	≥32 (R)	≥32 (R)	≥32 (R)	≥32 (R)	≥32 (R)	≥32 (R)
ampicillin/ sulbactam	NA	NA	NA	≥32 (R)	≥32 (R)	≥32 (R)	≥32 (R)	≥32 (R)	≥32 (R)	≥32 (R)	≥32 (R)
piperacillin/ tazobactam	≥128 (R)	≥128 (R)	≥128 (R)	≥128 (R)	≥128 (R)	≥128 (R)	≥128 (R)	≥128 (R)	≥128 (R)	≥128 (R)	≥128 (R)
cefazolin	≥64 (R)	≥64 (R)	≥64 (R)	≥64 (R)	≥64 (R)	≥64 (R)	≥64 (R)	≥64 (R)	≥64 (R)	≥64 (R)	≥64 (R)
cefepime	16 (R)	≥64 (R)	≥64 (R)	8 (SDD)	2 (SDD)	8 (SDD)	≥64 (R)	≥64 (R)	≥64 (R)	≥64 (R)	16 (R)
cefoxitin	≥64 (R)	≥64 (R)	≥64 (R)	≥64 (R)	≥64 (R)	≥64 (R)	≥64 (R)	≥64 (R)	≥64 (R)	≥64 (R)	≥64 (R)
ceftazidime	≥64 (R)	≥64 (R)	≥64 (R)	≥64 (R)	≥64 (R)	≥64 (R)	≥64 (R)	≥64 (R)	≥64 (R)	≥64 (R)	≥64 (R)
ceftriaxone	≥64 (R)	≥64 (R)	≥64 (R)	≥64 (R)	≥64 (R)	≥64 (R)	≥64 (R)	≥64 (R)	≥64 (R)	≥64 (R)	≥64 (R)
ciprofloxacin	1 (R)	2 (R)	1 (R)	≤0.25 (S)	1 (R)	1 (R)	≤0.25 (S)	≤0.25 (S)	≤0.25 (S)	≤0.25 (S)	≤0.25 (S)
levofloxacin	4 (R)	4 (R)	1 (R)	≤0.12 (S)	1 (R)	1 (R)	≤0.12 (S)	≤0.12 (S)	≤0.12 (S)	≤0.12 (S)	≤0.12 (S)
meropenem	≥16 (R)	≥16 (R)	≥16 (R)	≥16 (R)	≥16 (R)	≥16 (R)	≥16 (R)	≥16 (R)	≥16 (R)	≥16 (R)	≥16 (R)
aztreonam	0.19 (S)	NA	NA	0.064 (S)	NA	NA	0.094(S)	0.125 (S)	0.19 (S)	0.016 (S)	NA
trimethoprim/ sulphonamide	≤20 (S)	≥320 (R)	≥320 (R)	≥320 (R)	≥320 (R)	≥320 (R)	≥320 (R)	≥320 (R)	≥320 (R)	≥320 (R)	≥320 (R)
tigecycline	≥8 (R)	NA	NA	NA	NA	1 (S)	≤0.5 (S)	≤0.5 (S)	NA	≤0.5 (S)	NA
ceftazidime/ avibactam	>256 (R)	>256 (R)	NA	NA	NA	>256 (R)	>256 (R)	>256 (R)	≥256 (R)	>256 (R)	NA
ceftolozane/ tazobactam	>256 (R)	>256 (R)	NA	NA	NA	NA	NA	NA	≥256 (R)	NA	NA
colistin	0.38	NA	0.125	0.38	0.38	0.75	0.5	0.5	0.5	0.125	0.38
polymyxin B	0.5	NA	0.38	0.38	0.5	0.5	0.5	0.75	1.5	0.25	NA
minocycline	NA	NA	NA	4 (S)	NA	NA	NA	NA	NA	NA	NA
nitrofurantoin	NA	NA	64 (I)	NA	≤16 (S)	64 (I)	NA	NA	NA	NA	NA

NA, not applicable; S, susceptible; SDD, susceptible dose-dependent; I, intermediate; R, resistant.

**Figure 1.**
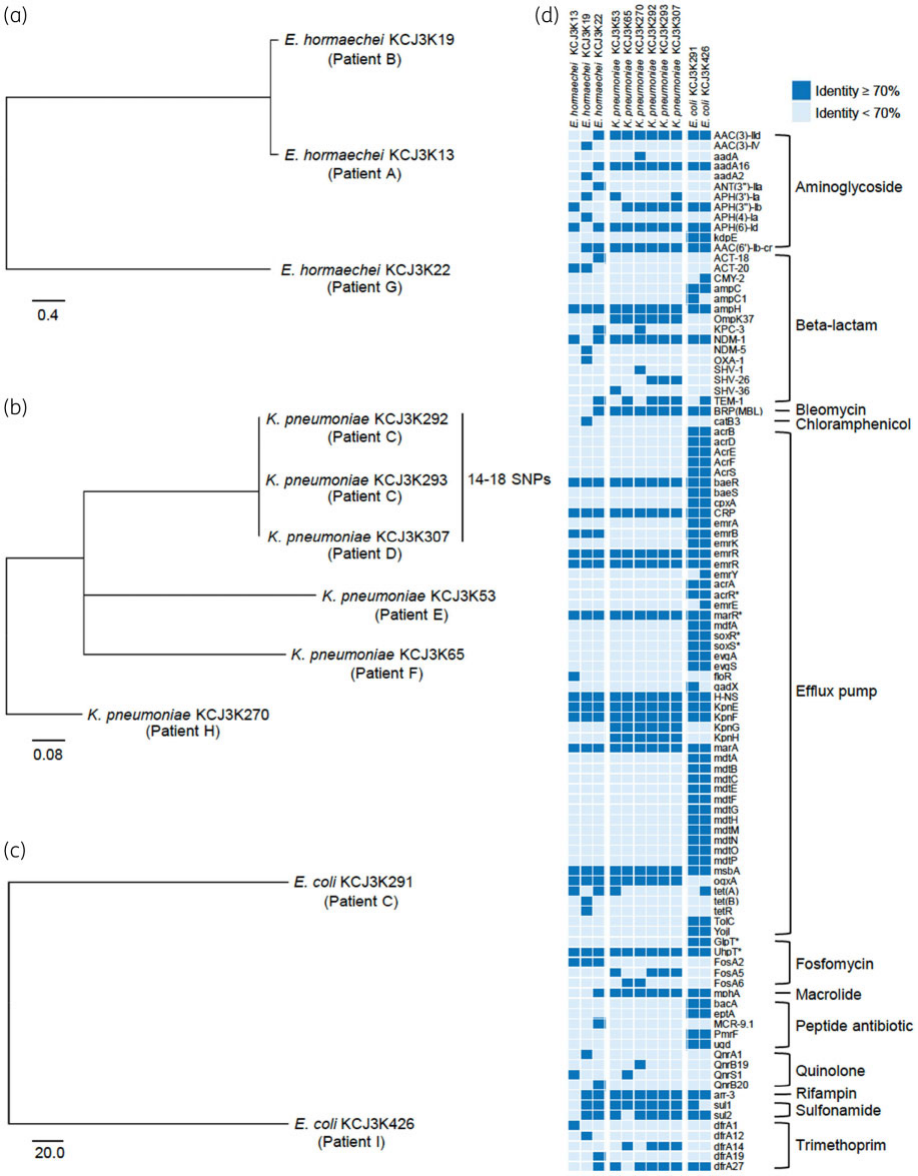
Genetic relatedness and ARG profile of the NDM isolates. The maximum-likelihood phylogenetic trees were constructed based on core-genome SNPs of Illumina sequencing data. Diverse *E*. *hormaechei* (a), *K. pneumoniae* (b) and *E. coli* (c) were isolated from patients. Clonal variants were isolated from different patients. (d) ARG profile. The ARGs of 11 strains were identified by comparing their genomic DNA sequences with CARD. The identified ARGs with more than 70% identity are shown in dark blue, while the ones with less than 70% identity are shown in light blue. The asterisks indicate the genes with mutations conferring antimicrobial resistance.

To investigate the genetic relatedness of the NDM isolates, WGS was conducted with the Illumina platform and subjected to a phylogenetic analysis. The phylogenetic trees were constructed for *E. hormaechei*, *K. pneumoniae* and *E. coli* (Figure 1a–c). Most isolates were genetically distinct. However, three *Klebsiella* strains (*K. pneumoniae* KCJ3K292, KCJ3K293, and KCJ3K307), isolated from patients C and D, were clonal variants with a difference of less than 18 SNPs in the genome. These data are consistent with transmission of this particular NDM-bearing bacterial strain among hospitalized patients.

With the CARD analysis, we identified 102 ARGs that are related to resistance to 12 classes of antibiotic. *E. coli* carried the highest number of ARGs (*n *=* *63 and 65), while *E. hormaechei* carried 23 to 34 ARGs and *K. pneumoniae* carried 30 to 33 ARGs. All isolates carried genes conferring resistance to aminoglycoside, β-lactam, fosfomycin, quinolone and trimethoprim, as well as genes encoding efflux pumps that confer MDR. Most of the identified ARGs were functional based on MIC (Table 1). Additionally, we identified genes conferring resistance to bleomycin, chloramphenicol, macrolide, peptide antibiotic, rifampicin and sulphonamide. Interestingly, *mcr-9.1*, the novel colistin resistance gene, was identified in *E. hormaechei* KCJ3K22, eliminating a key ‘last resort’ antimicrobial for these highly resistant strains, but this strain was susceptible to colistin (Table 1), likely due to low expression levels. Fortunately, this particular strain retained some activity to fluoroquinolones, which were used in combination in the treatment regimen for this patient.

To further dissect the dissemination mechanisms of NDM-bearing strains among hospitalized patients, we used the PacBio sequencing platform to close the genome for six isolates (*E. hormaechei* KCJ3K13 from patient A, *E. hormaechei* KCJ3K19 from patient B, *K. pneumoniae* KCJ3K292 from patient C, *K. pneumoniae* KCJ3K293 from patient C, *K. pneumoniae* KCJ3K307 from patient D, and *E. coli* KCJ3K291 from patient C); strains were selected based on close genetic distance to further characterize genetic variations and transmission patterns of NDM genes and NDM-bearing strains between patients. The six strains carried multiple plasmid types, including IncF family, IncR and IncX3 plasmids with a narrow host range of Enterobacteriaceae as well as IncHI2 and IncC plasmids with a broad host range (Table S3). The IncX3 plasmid carrying *bla*_NDM-1_ and *bla*_NDM-5_ was found in *E. hormaechei* strains, and the IncC plasmids carrying *bla*_NDM-1_ were identified in *K. pneumoniae* and *E. coli* strains (Table S3).

**Figure 2.**
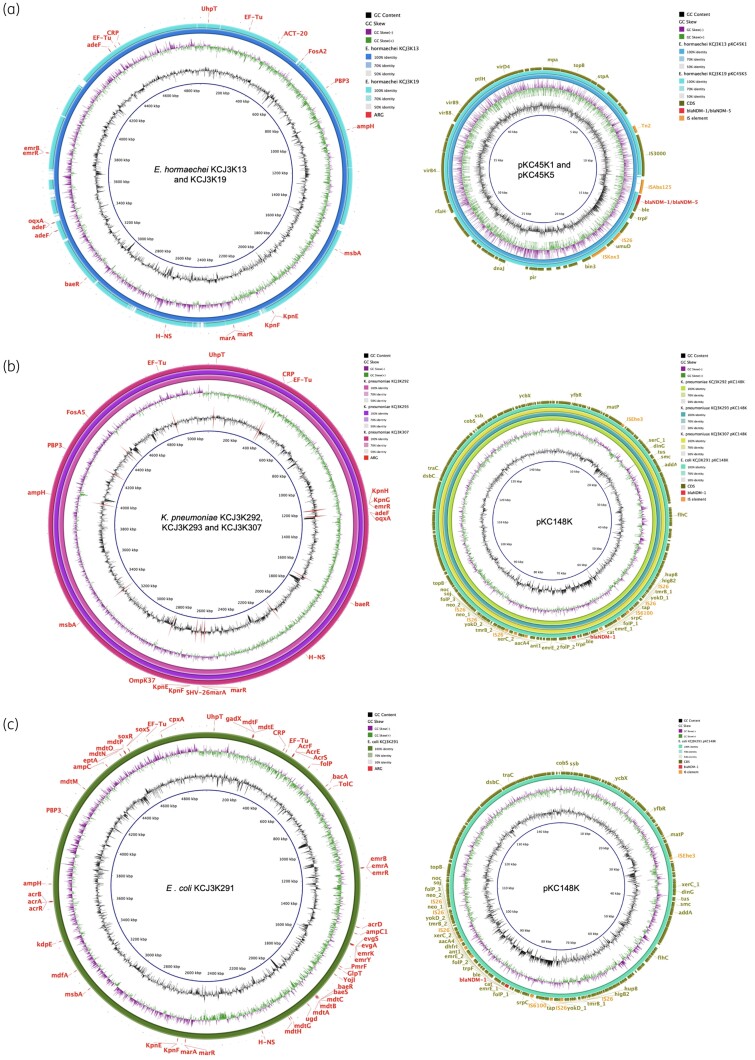
Chromosomal and plasmid genome maps of the NDM pathogens. Circular genome maps of chromosomal DNA and plasmids were generated using PacBio sequencing data. (a) *E. hormaechei.* (b) *K. pneumoniae*. (c) *E. coli*. The IncX3 plasmids, pKC45K1 and pKC45K5, were identical except for the *bla*_NDM_ genes and IS elements. The IncC plasmid, pKC148K, was harboured by three *K. pneumoniae* strains and one *E. coli* strain.

The chromosomes of *E. hormaechei* KCJ3K13 and KCJ3K19 had a similar genetic backbone, albeit with multiple insertion-deletions (indels) which clearly differentiated the two genomes. However, these two chromosomes carried the same 22 ARGs, such as β-lactamase-encoding genes *ACT-20* and *ampH* (Figure 2a). At the plasmid level, pKC45K1 carrying *bla*_NDM-1_ was identified in *E. hormaechei* KCJ3K13, while pKC45K5 carrying *bla*_NDM-5_ was identified in *E. hormaechei* KCJ3K19. These plasmids were identical except for a two amino acid difference between *bla*_NDM-1_ and *bla*_NDM-5_ and two IS elements, IS*Aba125* and IS*5*, which were located upstream of the *bla*_NDM_ genes (Figure 2a). This non-homologous IS region indicates that different IS-mediated transposition events occurred at the same loci of a plasmid carried by two different *E. hormaechei* strains. These two plasmids encode the VirB type IV secretion system that may transfer plasmids by conjugation.

The clonal variant *K. pneumoniae* strains isolated from two patients (KCJ3K292/KCJ3K293 from patient C; KCJ3K307 from patient D) carried 21 ARGs in the chromosome, including the ESBL-encoding gene *bla*_SHV-26_ and the AmpC-encoding *bla*_AmpH_ (Figure 2b). Based on the genetic identity, the *K. pneumoniae* KCJ3K307 might have originated from patient C or vice versa. Patient C was also infected with *E. coli* KCJ3K291, which carried 53 ARGs on the chromosome, including the β-lactamase-encoding genes *bla*_AmpC1_ and *bla*_AmpH_ (Figure 2c). Interestingly, the same IncC plasmid, pKC148K, encoding *bla*_NDM_, was harboured by *E. coli* KCJ3K291, *K. pneumoniae* KCJ3K292, *K. pneumoniae* KCJ3K293 and *K. pneumoniae* KCJ3K307, indicating that plasmid transmission probably occurred between two different genera in patient C, possibly mediated by the type IV conjugation system. pKC148K in the four strains was identical (≥99.99% identify, with 100% query coverage). The bacterial conjugation experiment confirmed that pKC148K could be transferred from *K. pneumoniae* KCJ3K292 to *E. coli* strain at frequencies ranging from 3 × 10^−4^ to 7.5 × 10^−4^ per recipient cell. The *bla*_NDM-1_ gene was located in a resistance island, carrying multiple resistance genes, *tmrB*, *ant1*, *neo* and *cat*. The resistance island carried five IS*26* elements and one IS*6100*, suggesting that multiple insertion events happened on this island. Additional plasmid, pKC141K was harboured by these *Klebsiella* isolates (Figure S1). Overall, we found that the *bla*_NDM_ gene was inserted into a broad host range conjugative plasmid that mediated transmission into different genera, indicating NDM transmission occurred within and between patients in the hospital.

**Figure 3.**
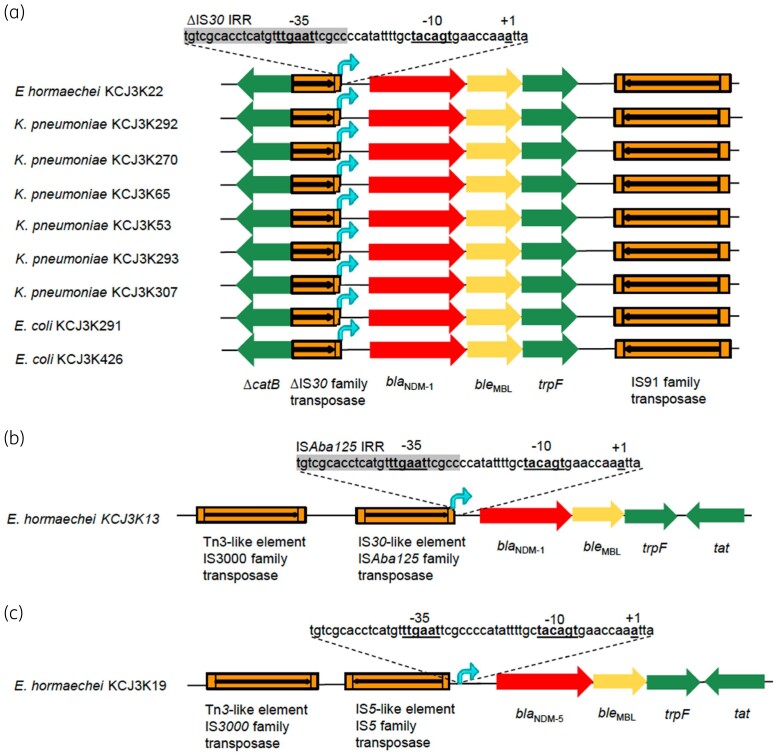
Transposition of *bla*_NDM_ genes by IS elements. Transposition types of *bla*_NDM_ were constructed by using Illumina sequencing data. (a) Mechanism 1 transposition. (b) Mechanism 2 transposition. (c) Mechanism 3 transposition. Three different ISs were identified upstream of the *bla*_NDM_ genes. *ble*MBL and *trp*F were identified downstream of *bla*_NDM_ in all 11 strains. Truncated IS*30* family transposase and intact IS*Aba125* provided the −35 box for *bla*_NDM-1_ transcription promoters. IS*5* was identified upstream of *bla*_NDM-5_ without involving in the promoter formation. Δ, truncated gene; IRR, right inverted repeats of IS element.

To further understand the role of IS elements on NDM expression, we investigated transcription mechanisms of the NDM genes. Three different transcription mechanisms were found in 11 NDM strains (Figure 3). Mechanism 1 was found in nine strains. A truncated IS*30* element provided the −35 region for the transcription of the *bla*_NDM-1_ gene, and the −10 region was provided by the NDM gene sequences (Figure 3a). The truncated IS*30* element was flanking a truncated *catB* gene, suggesting that an IS*30* element inserted into the *catB* gene. Similar to mechanism 1, mechanism 2 also used a hybrid promoter region: the −35 region was provided by the IS*Aba125* element, and the −10 box was provided by the NDM gene sequences (Figure 3b). In mechanism 3, IS*5* was located upstream of the *bla*_NDM-5_ gene, but the transcription promoter regions were fully coded by the NDM gene sequences (Figure 3c). Regardless of the genetic environment, *ble*_MBL_ and *trpF* were always identified downstream of the *bla*_NDM_ gene, indicating that these two genes were transferred together with the *bla*_NDM_ gene. Therefore, besides the *bla*_NDM_ gene mobilization, IS elements would appear to regulate the transcription of the gene.

**Figure 4.**
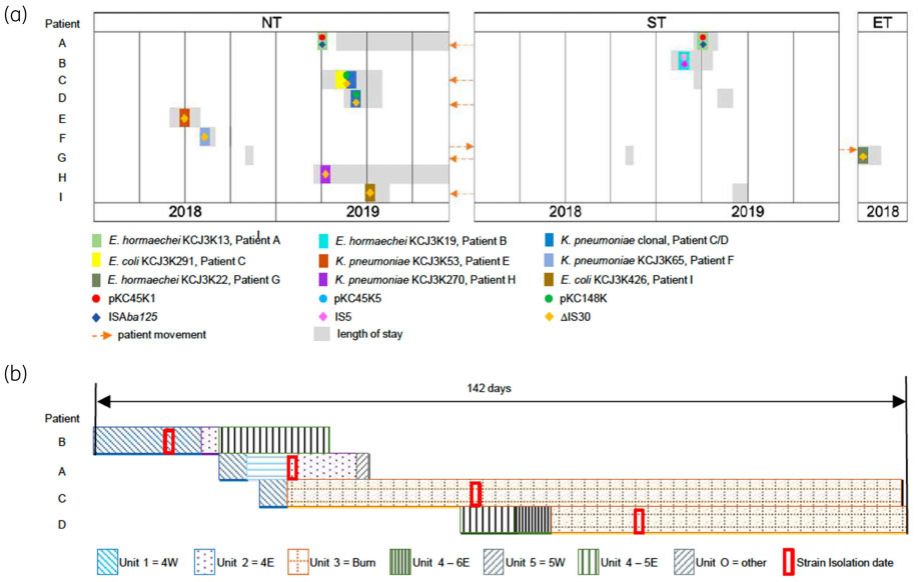
Schematic diagrams of patient movement and probable transmission of NDM strains, plasmids and IS elements in the hospital. Patient movement history in the hospital related to genetic features of the NDM-positive strains (a). Patients moved frequently between different facilities. Arrows indicate patient movement directions. The size of grey rectangle represents the length of hospital stay of individual patient. Coloured rectangles represent 12 NDM-positive strains isolated from the patients indicated. *K. pneumoniae* clonal variant was isolated from patient C and D. Coloured circles represent plasmids identified in the six strains shown as the rectangles. The identical plasmids, pKC45K1 and pKC45K5, were harboured in *E. hormaechei* KCJ3K13 and KCJ3K15, respectively. Plasmid pKC148K was identified in both *K. pneumoniae* clonal variant and *E. coli* KCJ3K291. Coloured diamonds represent different insertion sequences located upstream of *bla*_NDM_. NT, North Tower; ST, South Tower; ET, East Tower. (b). Schematic diagram of overlapped hospital stays at the units while patients B, A, C and D were hospitalized. Each patterned rectangle represents the length of stay and units. Red boxes indicate the dates of isolation of each NDM strain.

To better illustrate the potential dissemination events related to the *bla*_NDM_-carrying strains, a patient movement map was built, based on the clinical records and genetic characteristics of the isolates (Figure 4). Clonal variants—*K. pneumoniae* KCJ3K292, *K. pneumoniae* KCJ3K293 and *K. pneumoniae* KCJ3K307—were isolated from patients C and D who had a similar hospitalization history. Both of them stayed in the hospital ST for a short period, and then moved to the NT where they were present at the same time (Figure 4a). Patient C was negative for NDM-bearing organisms from initial wound cultures when the patient was admitted to Unit 1. However, 33 days after transfer to Unit 3, the patient was positive for NDM-bearing pathogens—*K. pneumoniae* KCJ3K292, *K. pneumoniae* KCJ3K293 and *E. coli* KCJ3K291—suggesting that patient C acquired these strains from Unit 3. To understand potential transmission routes of these strains, we conducted rectal screening and carbapenem resistance of all patients on Unit 3, but none of the patients was positive for NDM-1. However, 28 days later, the clonal variant strain *K. pneumoniae* KCJ3K307 was isolated from patient D in Unit 3 (Figure 4b). Patients C and D were in rooms next to one another, and the clonal variant strain was isolated 16 days after patient D admitted into Unit 3, suggesting NDM-producing *K. pneumoniae* was transmitted between the two patients during their hospital stay indirectly. However, environmental cultures obtained to understand possible transmission routes, including surfaces inside and outside the patient rooms, sink, the nurses’ station, equipment and operating room, were negative for any NDM-bearing or carbapenem-resistant organism. Besides *K. pneumoniae*, NDM-positive *E. coli* KCJ3K291 was also isolated from patient C. The *bla*_NDM-1_-carrying IncC plasmid, pKC148K, was harboured by *K. pneumoniae* clonal variants and *E. coli* KCJ3K291, suggesting the plasmid was transferred between different genera in patient C. However, *E. coli* bearing NDM-1 was not isolated in patient D. In addition, the same plasmids, pKC45K1 and pKC45K5, were isolated from patients A and B but we could not identify an epidemiological connection that might explain plasmid transmission between two patients.

## Discussion

In this study we characterized 11 MDR NDM-bearing pathogens from nine patients hospitalized in a single hospital during a 1 year time period. We found multiple genetic mechanisms involved in NDM transmission including clonal strain transmission, horizontal gene transfer mediated by plasmid conjugation and transposition by IS elements. With multiple antibiotic resistance determinants and various dissemination mechanisms, selection and transmission of NDM is complicated in the hospital setting. Connecting the genetic features of NDM-bearing strains with patient-movement history helped us link possible transmission mechanisms of the NDM genes.

Plasmids can contribute to the spread and evolution of ARGs.^12^ The result of plasmid typing showed that the *bla*_NDM_ genes were carried by IncX3 or IncC type plasmids (Table S3). As the most common *bla*_NDM_ gene carrier, IncX3 plasmids have been reported carrying multiple *bla*_NDM_ variants, raising the possibility that the *bla*_NDM_ gene evolved on IncX3 plasmids.^9^ The NDM-encoding plasmids, pKC45K1 and pKC45K5, harboured by *E. hormaechei* KCJ3K13 and KCJ3K19, respectively, are identical, except for the *bla*_NDM_ variants, *bla*_NDM-1_ and *bla*_NDM-5_ and IS elements. According to previous studies, NDM-5 confer greater resistance than NDM-1.^23^ In addition to the *bla*_NDM_ gene, the IncX3 plasmid is associated with *bla*_SHV-12_, *bla*_KPC_ and *bla*_OXA-181_.^24^^,^^25^ As a conjugative plasmid, IncX3 contributes to the horizontal gene transfer of the *bla*_NDM_ gene and other ARGs, and thus helps them spread among the Enterobacteriaceae. IncC is another common type of the *bla*_NDM_ gene-carrying plasmids.^9^ It has a broad host range, not only among Enterobacteriaceae, but also Morganellaceae and Vibrionaceae, contributing to the worldwide spread of *bla*_NDM_.^9^ Currently, a total of 20 replicon types have been reported in NDM-carrying plasmids in Enterobacteriaceae, suggesting *bla*_NDM_ can be acquired and transferred by multiple plasmids among strains, which makes it difficult to restrain the spread of *bla*_NDM_.^9^ In this study we found that the IncC plasmid could transfer to another patient and between *K. pneumoniae* and *E. coli.*

CRE usually carry multiple IS elements that affect neighbouring gene expression.^26^ The IncC plasmid, pKC148K, found in *K. pneumoniae* and *E. coli* carried five IS*26* and one IS*6100* on the resistance islands (Figure 2b and c), resulting in an MDR phenotype. Consistently, both IS*26* and IS*6100* belong to the IS*6* family and IS*26* has been reported associated with the *bla*_NDM_ gene transfer.^27^ There are two features commonly found next to the *bla*_NDM_ gene, IS elements and the bleomycin resistance gene *ble*_MBL_.^9^ IS elements were systematically found upstream of the *bla*_NDM_ gene providing the −35 box for transcription.^28^ Consistently, we identified IS elements in the upstream of the *bla*_NDM-1_ gene that initiate transcription. The *ble*_MBL_ gene is systematically found downstream of *bla*_NDM_, and expression of the *ble*_MBL_ gene is regulated by the same promoter regions. Bleomycin has been mostly used as an anticancer agent in clinical therapy, suggesting *bla*_NDM_-carrying strains might be selected by antibiotics or co-selected by bleomycin.^29^

Interestingly, all isolates tested against aztreonam displayed very low MIC with retained susceptibility. Though aztreonam is the only available β-lactam antibiotic stable to MBLs, it is susceptible to enzymatic degradation by ESBL and AmpC enzymes. However, all isolates from patients C and D retained *in vitro* susceptibility to aztreonam, despite encoding both ESBL and AmpC β-lactamases.

Our paper demonstrates the spread, persistence and complexity involved in movement of NDM resistance among patients in a hospital setting; it also highlights the utility of molecular analysis in understanding transmission mechanisms, and, in turn, in developing optimal control strategies for these pathogens. The demonstration of movement of resistant clonal strains among patients underscores the importance of optimizing ‘standard’ infection control procedures, particularly in high-risk settings where there are seriously ill patients with a history of prolonged and/or multiple recent hospital stays. Care should also be taken with use of empirical antimicrobial regimens, which have the potential for selecting out strains carrying complex resistance genes, and also encouraging the movement of mobile resistance elements (plasmids and IS elements) among strains. With the number of resistance determinants we have demonstrated in these NDM clinical strains, it is paramount to have robust antimicrobial stewardship efforts in place that control all classes of antimicrobials and the duration of their use.

## Supplementary Material

dlab032_Supplementary_Data
