# Supplementary material for: Dissemination mechanisms of NDM genes in hospitalized patients
Source: JAC Antimicrob Resist. 2021 Mar 30;3(1):dlab032. doi: 10.1093/jacamr/dlab032 (PMC8210240; doi:10.1093/jacamr/dlab032)
Supplement: dlab032_Supplementary_Data [file dlab032_supplementary_data.docx]

**Supplementary data**

| Strain | Accession Number |
| --- | --- |
| *E. hormaechei* KCJ3K13 | [SZNI00000000](https://www.ncbi.nlm.nih.gov/nuccore/SZNI00000000)  [CP054409-CP054410](https://submit.ncbi.nlm.nih.gov/api/2.0/files/jwnabjon/fused_sub.current.gb/?format=attachment) |
| *E. hormaechei* KCJ3K19 | [SZNJ00000000](https://www.ncbi.nlm.nih.gov/nuccore/SZNJ00000000)  [CP054411-CP054412](https://submit.ncbi.nlm.nih.gov/api/2.0/files/ggig1m5n/fused_sub.current.gb/?format=attachment) |
| *E. hormaechei* KCJ3K22 | [VOME00000000](https://www.ncbi.nlm.nih.gov/nuccore/VOME00000000) |
| *K. pneumoniae* KCJ3K53 | VTDV00000000 |
| *K. pneumoniae* KCJ3K65 | [VOMF00000000](https://www.ncbi.nlm.nih.gov/nuccore/VOMF00000000) |
| *K. pneumoniae* KCJ3K270 | [VOMG00000000](https://www.ncbi.nlm.nih.gov/nuccore/VOMG00000000) |
| *K. pneumoniae* KCJ3K292 | [VLIZ00000000](https://www.ncbi.nlm.nih.gov/nuccore/VLIZ00000000)  [CP054401-CP054403](https://submit.ncbi.nlm.nih.gov/api/2.0/files/342tdzsr/fused_sub.current.gb/?format=attachment) |
| *K. pneumoniae* KCJ3K293 | [VLJA00000000](https://www.ncbi.nlm.nih.gov/nuccore/VLJA00000000)  [CP054404-CP054406](https://submit.ncbi.nlm.nih.gov/api/2.0/files/wkpflyol/fused_sub.current.gb/?format=attachment) |
| *K. pneumoniae* KCJ3K307 | [VLJB00000000](https://www.ncbi.nlm.nih.gov/nuccore/VLJB00000000)  [CP054398-CP054400](https://submit.ncbi.nlm.nih.gov/api/2.0/files/dprd3oj1/fused_sub.current.gb/?format=attachment) |
| *E. coli* KCJ3K291 | [VOMI00000000](https://www.ncbi.nlm.nih.gov/nuccore/VOMI00000000)  [CP054407-CP054408](https://submit.ncbi.nlm.nih.gov/api/2.0/files/ucnivpdw/fused_sub.current.gb/?format=attachment) |
| *E. coli* KCJ3K426 | [VOMM00000000](https://www.ncbi.nlm.nih.gov/nuccore/VOMM00000000) |

**Table S1. Data Accession**

**Table S2: Antibiotic(s) treatment for NDM infection**

| Strains | Patient | Organism | Mechanism | Antibiotic(s) used to treat NDM infection | Specimen source |
| --- | --- | --- | --- | --- | --- |
| KCJ3K13 | A | *Enterobacter hormaechei* | NDM-1 | amikacin, aztreonam, ciprofloxacin | wound |
| KCJ3K19 | B | *Enterobacter hormaechei* | NDM-5 | Amikacin and aztreonam | wound |
| KCJ3K22 | G | *Enterobacter hormaechei* | NDM-1, KPC-3 | ciprofloxacin | urine |
| KCJ3K53 | E | *Klebsiella pneumoniae* | NDM-1 | amikacin, aztreonam, ciprofloxacin | blood |
| KCJ3K65 | F | *Klebsiella pneumoniae* | NDM-1 | amikacin | urine |
| KCJ3K270 | H | *Klebsiella pneumoniae* | NDM-1, KPC-3 | Unknown (result obtained after discharge) | urine |
| KCJ3K292 | C | *Klebsiella pneumoniae* | NDM-1 | amikacin and aztreonam | wound |
| KCJ3K293 | C | *Klebsiella pneumoniae* | NDM-1 | amikacin and aztreonam | wound |
| KCJ3K307 | D | *Klebsiella pneumoniae* | NDM-1 | aztreonam and tigecycline | wound |
| KCJ3K291 | C | *Escherichia coli* | NDM-1 | amikacin and aztreonam | wound |
| KCJ3K426 | I | *Escherichia coli* | NDM-1 | ciprofloxacin | ascitic fluid |
|  | | | | | |

**Table S3. Plasmid replicon types of NDM carrying strains**

| Strains | Circular Plasmids | Replicon Types |
| --- | --- | --- |
| KCJ3K13 | pKC45K1 | IncX3 ^a^ |
|  |  | IncR |
| KCJ3K19 | pKC45K5 | IncX3 ^a^ |
|  |  | IncHI2 |
|  |  | IncHI2A |
| KCJ3K291 | pKC148K | IncC ^a^ |
|  |  | IncFII |
| KCJ3K292 | pKC148K | IncC ^a^ |
|  |  | IncFIB(pQil) |
|  | pKC141K | IncFIB(K) |
|  |  | IncFII(K) |
|  |  | IncR |
| KCJ3K293 | pKC148K | IncC ^a^ |
|  | pKC141K | IncFIB(K) |
| KCJ3K307 | pKC148K | IncC ^a^ |
|  | pKC141K | IncFIB(K) |
|  |  | IncFIB(pQil) |
|  |  | IncFII(K) |
|  |  | IncR |
| ^a^ Replicon types of *bla*NDM carrying plasmids | | |


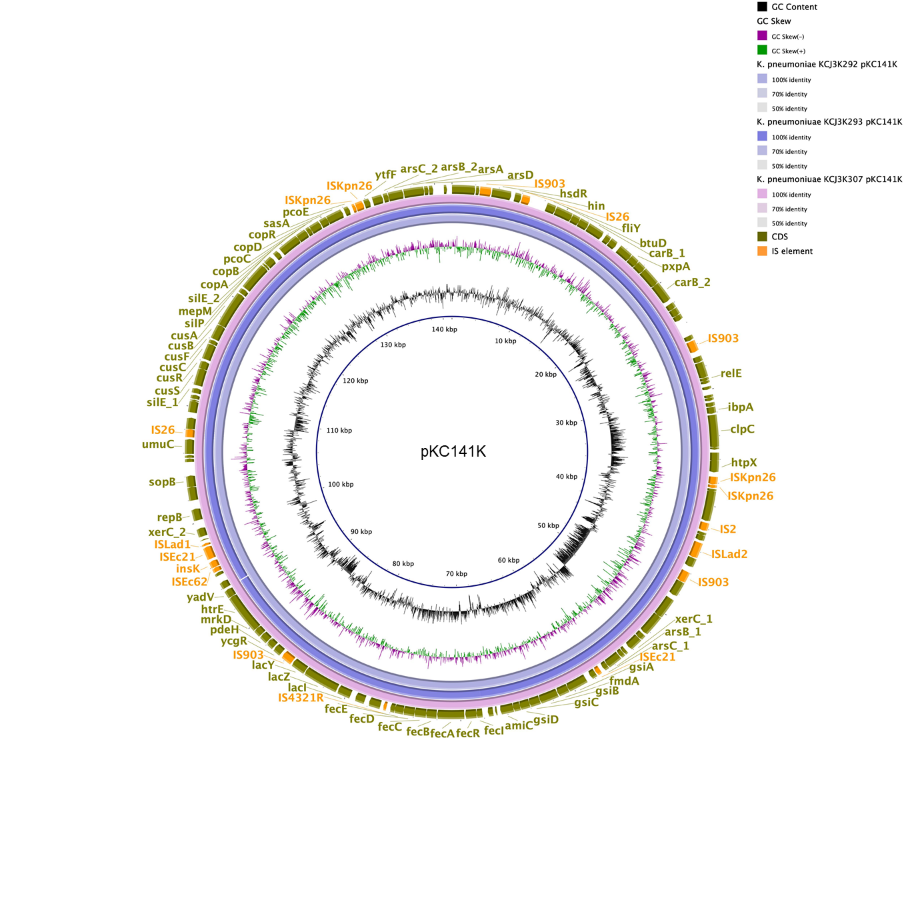


**Figure S1: pKC141K genome map**

Circular genome map of plasmid pKC141K, identified in three *K. pneumoniae* strains, KCJ3K292, KCJ3K293 and KCJ3K307, isolated from different patients.
